# A modified approach for programmed electrical stimulation in mice: Inducibility of ventricular arrhythmias

**DOI:** 10.1371/journal.pone.0201910

**Published:** 2018-08-22

**Authors:** Lukas Clasen, Christian Eickholt, Stephan Angendohr, Christiane Jungen, Dong-In Shin, Birgit Donner, Alexander Fürnkranz, Malte Kelm, Nikolaj Klöcker, Christian Meyer, Hisaki Makimoto

**Affiliations:** 1 Division of Cardiology, Pulmonology and Vascular Medicine, Medical Faculty, University Duesseldorf, Duesseldorf, Germany; 2 Cardiovascular Research Institute Duesseldorf (CARID), University Duesseldorf, Medical Faculty, Duesseldorf, Germany; 3 Department of Pediatric Cardiology and Pneumology, University Children's Hospital, Duesseldorf, Germany; 4 Institute of Neuro- and Sensory Physiology, Medical Faculty, Heinrich-Heine-University, Duesseldorf, Germany; University of Minnesota, UNITED STATES

## Abstract

**Background:**

Electrophysiological studies in mice, the prevailing model organism in the field of basic cardiovascular research, are impeded by the low yield of programmed electrical stimulation (PES).

**Objective:**

To investigate a modified approach for ventricular arrhythmia (VA) induction and a novel scoring system in mice.

**Method:**

A systematic review of literature on current methods for PES in mice searching the PubMed database revealed that VA inducibility was low and ranged widely (4.6 ± 10.7%). Based on this literature review, a modified PES protocol with 3 to 10 extrastimuli was developed and tested in comparison to the conventional PES protocol using up to 3 extrastimuli in anesthetized wildtype mice (C57BL/6J, n = 12). Induced VA, classified according to the Lambeth Convention, were assessed by established arrhythmia scores as well as a novel arrhythmia score based on VA duration.

**Results:**

PES with the modified approach raised both the occurrence and the duration of VA compared to conventional PES (0% vs 50%; novel VA score p = 0.0002). Particularly, coupling of >6 extrastimuli raised the induction of VA. Predominantly, premature ventricular complexes (n = 6) and ventricular tachycardia <1s (n = 4) were observed. Repeated PES after adrenergic stimulation using isoprenaline resulted in enhanced induction of ventricular tachycardia <1s in both protocols.

**Conclusion:**

Our findings suggest that the presented approach of modified PES enables effective induction and quantification of VA in wildtype mice and may well be suited to document and evaluate detailed VA characteristics in mice.

## Introduction

Mice present the prevailing model organism in basic cardiovascular research. They play a key role in the translation of molecular and cellular mechanisms into the *in-vivo* setting [1]. This is of increasing relevance in the ongoing exploration of cardiac arrhythmia mechanisms and the quest for more effective antiarrhythmic treatment options [2].

There is consensus on the definition of murine arrhythmias summarized in the revised Lambeth Convention [3]. The idea of employing a scoring system for ventricular arrhythmias (VA) was developed first in 1988 and was recently taken up again [4–6]. It could serve as a reference for interpreting electrophysiological findings in murine models of diseases.

However, murine vulnerability for VA has been discussed ambiguously, especially in comparison to humans. The long-term accepted critical mass theory presented by Garrey [7] has been questioned in this context, as sustained VA could be shown in <1 cm^2^ tissue areas [8]. In the current literature, inducibility of VA in C57BL/6 wildtype mice is mostly limited to short runs of VA when using classical programmed electrical stimulation (PES) protocols.

It is well known that the majority of VA results from triggered activity or re-entry [9, 10]. Arrhythmias by re-entrant mechanisms may well be induced by a critical delay in the re-entrant circuit by a greater number of extrastimuli [10]. Arrhythmias by triggered activity, particularly delayed afterdepolarization, are efficiently induced by Ca^2+^-overload by a greater number of extrastimuli [11–13]. Therefore, we herein hypothesized that the inducibility of VA in wildtype mice is facilitated by increasing the number of ventricular extrastimuli in PES.

Our findings in the present study suggest that a modified approach of PES in mice is useful to efficiently document and evaluate acutely induced VA.

## Materials and methods

### Systematic literature review

A systematic literature research of the PubMed database was conducted using the search terms: “in vivo cardiac electrophysiology AND mouse”, “intracardiac electrophysiology AND mouse”, “mouse electrophysiology AND ventricular arrhythmia”, “in vivo ventricular arrhythmia AND mouse” and “programmed electrical stimulation AND mouse”, for the period from 1 January 1996 until 1 January 2017. In an attempt to access the grey literature, we searched the OpenGrey Database (http://www.opengrey.eu). Only in vivo electrophysiological study (EPS) with jugular transvenous approach and ventricular programmed stimulation were considered. We excluded references with VA induction solely by ventricular burst-stimulation and not published in English unless an English version of the abstract was accessible.

### Animal care

Animal care, all experiments and euthanasia were conducted adhering to established guidelines [14] and with the prospective approval of the North Rhine-Westphalian State Agency for Nature, Environment and Consumer Protection (LANUV, permit number: G460/13). In this study male wildtype mice C57BL/6J (n = 12; 14–16 weeks; 25.5 ± 2.5g) were held on standard chow and tap water ad libitum, under a 12/12 hours light/dark cycle [15]. All experiments were conducted under mild sedation with isoflurane 1.6 Vol.% (induction period 2.5 Vol.% in 70% N2O / 30% O2), monitored by hind limb reflex and controlled by spontaneous breathing rate. Body core temperature was strictly maintained continously at 36°C via a rectal probe (T-type Pod ML312; Rectal Probe for Mice RET-3, ADInstruments) (**S1 Fig, S1 Text**). Duration of experiments was limited to 90 minutes to avoid sedation overload [16]. Euthanasia was conducted at the end of the study by exsanguination after deep anesthesia with intraperitoneal administration of 100mg/kg body-weight ketamine and 10mg/kg body-weight xylazine.

Cardiac function was assured in a subgroup of tested mice by cardiac echocardiography (**S1 Table, S2 Text**).

### Electrophysiological study and ventricular PES

Surface ECG was obtained in Einthoven lead I using clamp electrodes (ECG amplifier, Hugo Sachs) connected to a PowerLab 8/30 (ADInstruments). Endocardial EPS was conducted using a 2.0 F (0.67mm) octapolar electrophysiology catheter (0.5mm electrode spacing; CIBer Mouse, NuMed) as described earlier [17, 18]. The catheter was positioned into the right ventricle via the right jugular vein under electrocardiographic monitoring. Electrodes 1–4 sensed ventricular activity, electrodes 5–6 recorded near the His region and electrodes 7–8 detected atrial activity. Data was analyzed with the LabChart 7.3.7 software suite (ADInstruments). Measurements comprised heart rate (HR, bpm), cycle length (CL, ms), P-wave duration (ms), PR interval (ms), QRS interval (ms) and rate corrected QT interval (ms) [19].

**Fig 1:**
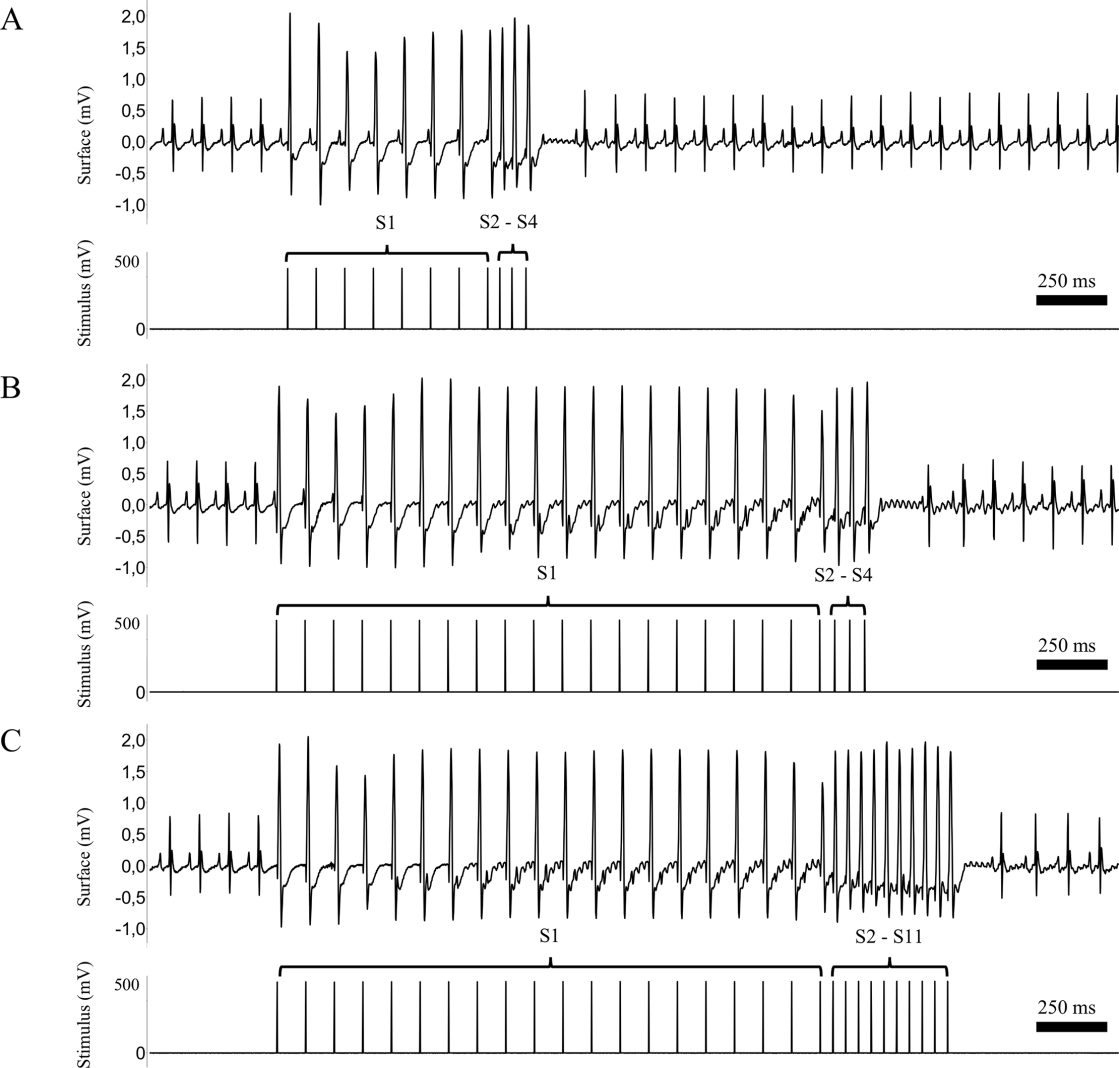
Examples of conventional and modified protocols to investigate the inducibility of ventricular arrhythmias by programmed electrical stimulation. A: Conventional programmed electrical stimulation (PES) protocol, which consists of eight S1 stimuli followed by two (S3) to three (S4) extrastimuli that are coupled in decrements of 2ms intervals from 60ms to 20ms. B: A representative example of the modified extrastimulation protocol depicts twenty S1 stimuli followed by three (S2-S4) extrastimuli. This protocol was defined as “miniburst (MB)”. C: The miniburst protocol with ten (S2-S11) extrastimuli. (for details see text).

Ventricular stimulation was achieved by rectangular impulses of 1 ms delivered at twice the pacing threshold voltage. Ventricular refractory period (VRP) was obtained by S1S2 stimulation with trains of 8 S1-stimuli (CL 100ms, 90ms, 80ms) and decremental coupling of S2 intervals (initial coupling interval 60ms, 2ms decrement) [18]. To induce two protocols were used, based on the literature review (**S2 Fig**):

1. Modified burst-stimulation protocol (miniburst, MB): Three to ten (S2S3S4—S11) extrastimuli after a train of twenty S1-stimuli (CL 100ms). Extrastimuli were coupled at an interval from 60 to 20ms with 2ms decrements (**Fig 1B** and **1C**).
2. Conventional PES protocol: Two (S2S3) and three (S2S3S4) extrastimuli (initial coupling interval 60ms, 2ms decrement) after a train of eight S1-stimuli (CL 100ms) [17, 18]. When coupling two extrastimuli the first extrastimulus was delivered at 20ms above the refractory period and the second extrastimulus was coupled at an interval from 60 to 20ms with 2ms decrements. When coupling three extrastimuli the first and second extrastimuli were delivered at 20ms above the refractory period and the third extrastimulus was coupled at an interval from 60 to 20ms with 2ms decrements. (**Fig 1A**).

Decremental pacing until 20ms was performed in order to ensure passing the refractory period. There was a period of recreation in sinus rhythm allowed for at least 2s between each pacing step. The 2 second-interval between each stimulation episode was adopted from the human electrophysiological test, because our literature review revealed that data on Ca^2+^ decay after stimulation episodes in mice were scarce [13, 20].

To assess individual VA inducibility after sympathetic activation each protocol was repeated with isoprenaline (1μg/g i.p.), resulting in an HR increase of at least 10% of baseline HR [18]. Direct progression to S11-stimulation was conducted during the MB protocol without MB S4 –S10, to avoid a prolonged study. All mice received exactly the same protocol.

### Arrhythmia assessment

**Fig 2:**
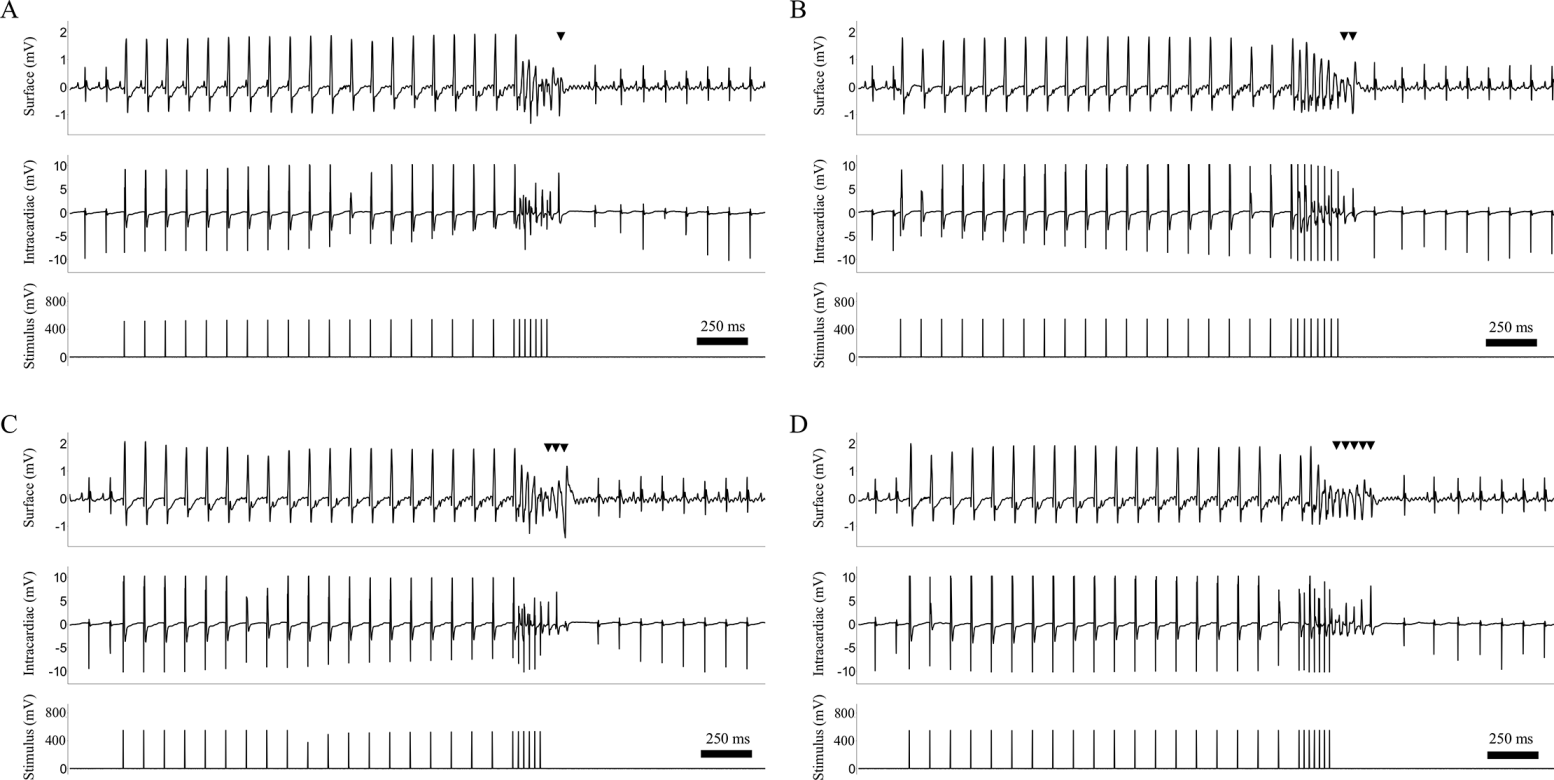
Systematic classification of ventricular arrhythmias based on the Lambeth criteria. Four types of ventricular arrhythmias are defined by international guidelines (11). A single premature ventricular complex (A), a ventricular couplet (B), triplet (C) and a ventricular tachycardia (D). Vertical arrow heads assign each premature ventricular complex. The horizontal square brackets depict programmed electrical stimulation. Surface electrocardiogram Einthoven lead I, intracardiac bipolar electrogram from mid RV and stimulus output are displayed.

VA induced during EPS were analyzed according to international consensus [3], using two established arrhythmia scores (Faggioni et al = score 1; van der Werf et al = score 2) [5, 21]. The score by Faggioni et al. bases on the worst ventricular arrhythmia displayed: 0 for no ventricular premature beats (VPBs) or isolated VPBs; 1 for frequent/bigeminal VPBs; 2 for couplets; and 3 for ventricular tachycardia. The arrhythmia score by Van der Werf et al is defined by the worst ventricular arrhythmia: 1 = no or isolated VPBs, 2 = bigeminal VPBs and/or frequent VPBs (>10 per minute), 3 = couplet, 4 = non-sustained VT (NSVT; ≥3 successive VPBs). Additionally, a novel arrhythmia score (score 3) was developed, differentiating VA by duration by assigning increasing score values (**Fig 2**): single premature ventricular complex (PVC) (1 point), couplet (3 points), triplet (4 points) and ventricular tachycardia (VT) (≥4 ventricular complexes = 5 points, ≧1s = 6 points) (**Table 1**).

**Table 1:** Scoring systems of ventricular arrhythmias.

	Score 1	Score 2	Score 3
PVC	0	1	1
Bigemini	1	2	-
Couplet	2	3	3
Triplet	3	4	4
VT < 1s	3	4	5
VT > 1s	3	4	6

Arrhythmia scores reflect the occurence of ventricular arrhythmias (VA). Each type of VA is assigned with fixed score points. Compared to gold standard arrhythmia scores by Faggioni et al. (Score 1) and van der Werf et al. (Score 2) a novel score (Score 3) was adapted for induced VA in mice. Bigemini as well as ventricular fibrillation did not occur after programmed electrical stimulation in the present studies. PVC = premature ventricular complex, VT = ventricular tachycardia.

### Statistical analysis

Statistical analysis was performed with Prism for Windows (V6.0; Graph Pad Software). Data are shown as mean ± SEM. Nonparametric data were analyzed using the Kruskal-Wallis test with Dunn’s multiple comparison. Arrhythmia score data were analyzed using the Wilcoxon matched-pairs signed rank test. Comparison of arrhythmia scores were analyzed using the two-way ANOVA Tukey´s multi comparison test. Continuous variables were evaluated using unpaired t-test. P<0.05 was considered statistically significant.

## Results

### Systematic review of literature

The initial PubMed search yielded 659 results of which 42 met the predefined selection criteria (**Table 2**). Altogether, 435 mice of different genetic backgrounds were included (C57 n = 138, FVB n = 66, 129 n = 22, CD1 n = 6, C3H n = 5, mixed background n = 142, unspecified n = 56).

**Table 2:** Results of systematic literature research concerning in vivo programmed ventricular stimulation in mice.

Protocol	Reference	Year	Strain	Pharmacological Provocation	Inducibility (%)
PES two extrastimuli	Berul Sidhu Garcia-Gras Lu Wagner Sawaya Nabben	1998 2005 2006 2006 2006 2007 2014	129/BS; 129SvEv FVB 129/SvJ C57/FVB C57/BS ICR/C57Bl/6 C57Bl/6	Isoprenaline 1–3 ng/g/min Procainamide none Epinephrine none none none	0 0 0 0 0 0 22.2
PES three extrastimuli	Berul Berul Bevilacqua Bevilacqua Jeron Gehrmann Berul Gehrmann Donoghue Kodirov Saba Ouvrard-Pascaud Zhang Wolf Korte Kannankeril Song Wolf Li Shusterman Le Quang Ye Leroy Bun Petric Yasuno Westphal Levin Petric	1996 1997 1999 1999 2000 2001 2001 2002 2003 2004 2004 2005 2005 2005 2005 2006 2007 2008 2009 2010 2011 2011 2011 2012 2012 2013 2013 2015 2016	C57Bl/6 129/BS not specified not specified FVB FVB 129/BS, 129SvEv FVB FVB FVB/C57Bl/6 C3H not specified (mixed) C57Bl/6 129SvEv C57Bl6 sham op C57Bl/6 C57Bl/6 not specified C57Bl/6 FVB C57Bl/6/SV129 CD1 or C57Bl/6 Ola/C57Bl/6 C57Bl/6J C57Bl/6 C57Bl/6 C57BL/6NTac C57Bl/6 C57Bl/6/129SV	Procainamide + Quinidine Isoprenaline 1–10 ng/g/min Isoprenaline 1 ng/g Isoprenaline 1 ng/g Isoprenaline 30μg Sham operated Isoprenaline 1–3 ng/g Carbachol none Atropine + Isoprenaline 10 μg Atropine + Propranolol none Carbachol none none Isoprenaline 100 μg none none Carbachol Isoprenaline 1 μg none Isoprenaline 2.5 μg/g Isoprenaline 100 nM none Isoprenaline 1 μg/g none none none Isoprenaline 1 μg/g	0 0 6.25 0 7 0 0 0 0 10 0 14 0 0 0 0 0 0 0 0 0 0 0 0 0 0 14 0 0
PES four extrastimuli	Rakhit Constantini Zuberi	2001 2005 2010	129/BS CD1 129SvEv	Isoprenaline 1 ng/g none Isoprenaline 0.1 μg/g	20 0 16.7
PES five extrastimuli	Breckenridge Sastry	2009 2006	CBA/Black10 C57Bl/6	Isoprenaline 0.1 μg/g none	28.6 0
PES ten extrastimuli	Prestia	2011	C57Bl/6 sham op	none	55

The PubMed data base was screened for in vivo transvenous PES in healthy wildtype mice. PES protocol, mouse strain and pharmacological stimulation as stated. VA induction varies independent of medical provocation from 0 to 55%. PES = programmed electrical stimulation.

The number of coupled extrastimuli during PES varied among studies. Double extrastimuli (S1S2-S3) were used in only seven studies. A majority of 29 studies performed double (S1S2-S3) and triple extrastimuli (S1S2-S4). Four studies coupled four extrastimuli (S1S2-S5), one coupled five extrastimuli (S1S2-S6), one coupled six extrastimuli (S1S2-S7) and one added up to ten extrastimuli (S1S2-S11) in case three extrastimuli did not yield VT.

In most studies, the definition of VA was different from the current guidelines (Lambeth convention), used in the present study, rendering a direct comparison of those studies unfeasible. Some studies counted only VTs >4 [22, 23], or >10 consecutive beats as VA [24] while others regarded single PVCs as VA [25]. Mean VA inducibility by PES was 4.6 ± 10.7% (20/435 mice, 10/42 studies), and varied with the number of extrastimuli: Two extrastimuli induced VA in 2.8% (2/71 mice, 1/7 studies) [26] and three extrastimuli induced VA in 2.5% (8/314 mice, 6/30 studies). Four to six extrastimuli induced VA in 12.2% (6/49 mice, 3/5 studies). Three to ten extrastimuli resulted in 55% VA induction (6/11 mice, 1 study) [27]. These results indicate a correlation between the number of extrastimuli and VA inducibility. Inducibility of VA was similar across all mouse strains tested (C57 6.5%, FVB 4.5%, 129 5.6% and mixed background 4.5%).

It was difficult to interpret the impact of isoprenaline on arrhythmia inducibility due to the differences in stimulation protocols and VA definitions. In 11 studies, the incidence of VA induction was 8.3% (8/96) after administration of isoprenaline as compared to 3.0% (3/100) under baseline conditions.

### Electrophysiological study

The data recorded during the present study are presented in **S1 Dataset**. Electrophysiological properties recorded by surface and intracardiac ECG were in accordance with previously published data [28, 29]: QRS width was 10.4 ± 1.2 ms, QT was 57.1 ± 7.1 ms, and QTc was 50.7 ± 5.8 ms. VRP ranged from 32.2 ± 6.9 ms (S1 100ms) to 34.0 ± 7.5 ms (S1 80ms) at baseline. Baseline sinus CL was 112.5 ± 7.8 ms, decreasing to 100 ± 4.3 ms (quotient 12.4%, HR 535.7 ± 33.1 bpm to 601.3 ± 25.6 bpm p<0.0001) after isoprenaline administration (**Table 3, S3 Fig**).

**Table 3:** Electrophysiological baseline characteristics.

Electrophysiological properties	
n	12
Weight (g)	25.5 ± 2.5
Temperature (°C)	35.9 ± 0.5
Heart Rate (bpm)	535.7 ± 33.1
Cycle Length (ms)	112.5 ± 7.8
Heart Rate Isoprenaline (bpm)	601.3 ± 25.6
Cycle Length Isoprenaline (ms)	100.0 ± 4.3
PR (ms)	38.3 ± 3.4
QRS (ms)	10.4 ± 1.2
QT (ms)	57.1 ± 7.1
QTc (ms)	50.7 ± 5.8
JT (ms)	46.7 ± 6.4
Ventricular threshold (mV)	225 ± 72
VRP 100 (ms)	32.0 ± 6.9
VRP 90 (ms)	32.8 ± 6.6
VRP 80 (ms)	34.0 ± 7.5

Electrophysiological (EP) baseline parameters for animals undergoing EP testing (n = 12). VRP = ventricular refractory period

**Fig 3:**
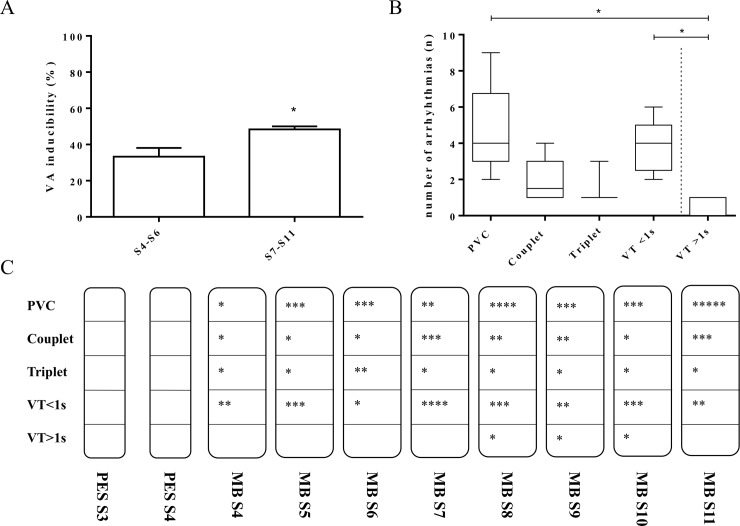
Ventricular arrhythmia induction with the miniburst protocol. Modified PES (MB) yielded VA in up to 50% of tested animals (n = 12). Inducibility increased when coupling of >6 extrastimuli (S4-S6 vs S7-S11 p = 0.011) (A). Predominantly, PVCs or VT of a duration less than 1 sec. were seen. PVC vs VT>1s p = 0.031, VT<1s vs VT>1s p = 0.030, Couplet vs VT>1s p = 0.096. Data plot as box and whiskers (B). Qualitative display of the number of extrastimuli in correlation with arrhythmia type. Quantity and duration of VA increased when coupling 7, 8 and 10 extrastimuli by means of ventricular events (MB S8 vs PES S2S3/S2S3S4 p = 0.011, MB S9 vs PES S2S3/S2S3S4 p = 0.025, MB S10 vs PES S2S3/S2S3S4 p = 0.312, MBS11 vs PES S2S3/S2S3S4 p = 0.0002). Stars represent animals affected (C).

Under baseline conditions no VA induction was observed with the conventional PES protocol. Using the MB protocol, however, VA were induced in 50% of tested mice. Quantity and duration of VA increased with coupling of >6 extrastimuli (Two-way ANOVA, and Tukey’s multiple comparison as post-hoc analysis: MB S4 vs PES S2S3/S2S3S4 p = 0.909, MB S5 vs PES S2S3/S2S3S4 p = 0.312, MB S6 vs PES S2S3/S2S3S4 p = 0.105, MB S7 vs PES S2S3/S2S3S4 p = 0.054, S8 vs PES S2S3/S2S3S4 p = 0.011, MB S9 vs PES S2S3/S2S3S4 p = 0.025, MBS10 vs S2S3/S2S3S4 p = 0.312, MBS11 vs PES S2S3/S2S3S4 p = 0.0002) (**Fig 3**).

**Fig 4:**
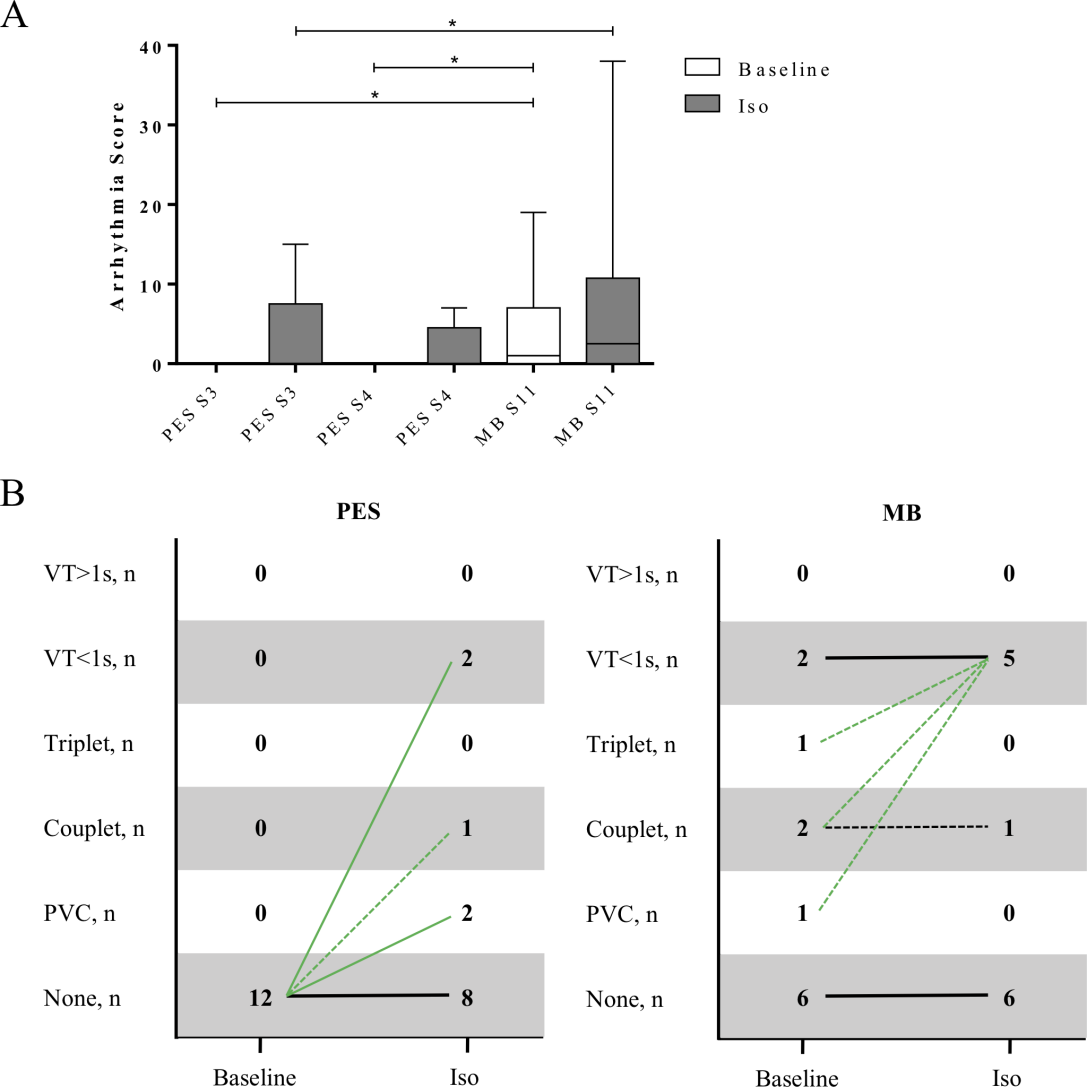
Impact of isoprenaline on ventricular arrhythmia induction. PES in relation to inducibility of VA before and after isoprenaline injection (Baseline: MB S11 vs PES S3/S4 p = 0.031; Iso: S3 vs S11 p = 0.031, S4 vs S11 p = 0.063). Data plot as box and whiskers (A). Qualitative VA depiction of affected mice, only most severe ventricular event shown. Shown left results of conventional PES S2S3S4 (p = 0.063) and right MB S11 (p = 0.281). Green line indicates increase of VA. Thick line indicates ≥3 mice, thin line 2 mice, dotted line 1 mouse (B). Iso = isoprenaline, n = 12.

Under isoprenaline, VA were induced using both protocols; yet the increase of VA compared to baseline control was not significant (PES S2S3 baseline none vs 4 PVCs, 3 couplets, 1 triplets, 4 VTs<1s, 0 VT>1s in 4 mice after isoprenaline, arrhythmia score 3, p = 0.125; PES S2S3S4 baseline none vs 7 PVCs, 2 couplets, 0 triplet, 2 VTs<1s, 0 VT>1s in 5 mice after isoprenaline, arrhythmia score 3, p = 0.063; MB: baseline 9 PVCs, 4 couplets, 1 triplet, 5 VTs<1s, 0 VT>1s in 6 mice vs 9 PVCs, 5 couplets, 3 triplets, 10 VTs<1s, 0 VT>1s in 5 mice after isoprenaline, arrhythmia score 3, p = 0.281) (**Fig 4**).

Nonetheless, also under isoprenaline stimulation, the MB protocol was more effective in provoking VA compared to conventional PES protocols (MB S11 vs PES S2S3/S2S3S4, arrhythmia score 3 p = 0.031/p = 0.063) (**Fig 4**). Particularly, VT induction was more effective when comparing the MB protocol to the conventional PES protocol with three extrastimuli (MB S11 10 VT<1s in 5 mice vs PES S2S3 4 VT<1s in 2 mice p = 0.172, MB S11 vs PES S2S3S4 2 VT<1s in 2 mice p = 0.049). In addition to 2 mice in which VT<1s was inducible without Iso, VT<1s was inducible in 3 mice in which PVC, couplet and triplet were induced by MB protocol without Iso. This result indicates that the VA inducibility by the MB protocol without Iso may reflect arrhythmogenic properties mainly related to triggered activity in these mice. Thus, sensitivity of the MB protocol without Iso appears higher (PES 0% [0/5] vs MB 100% [6/6]) as compared to the PES protocol without Iso. Under baseline conditions, 6 mice without VA inducibility by MB did not show VA inducibility under Iso. This may indicate higher negative predictive value of baseline MB protocol (PES 58% [7/12] vs MB 100% [6/6]). In the present study, specificity of both baseline protocols was 100% (PES 7/7 vs MB 6/6).

### Arrhythmia assessment

Cumulative events of recorded VA ranged from single PVCs (n = 38), couplets (n = 16), triplets (n = 10) to VTs <1s (n = 32) and VT >1s (n = 3). The number of animals with PVC and VT <1 s was 6 and 4, respectively (**Fig 3B**).

Application of the MB protocol induced VA already under baseline conditions. When applying arrhythmia score 1, MB extrastimulation resulted in 11 (S4), 17 (S5), 14 (S6), 27 (S7), (S8), 27 (S9), 20 (S10) and 26 (S11) cumulative score points. With arrhythmia score 2, MB extrastimulation resulted in 17 (S4), 27 (S5), 25 (S6), 40 (S7), 37 (S8), 41 (S9), 30 (S10) and 45 (S11) cumulative score points. According to arrhythmia score 3, MB extrastimulation resulted in 19 (S4), 31 (S5), 26 (S6), 46 (S7), 43 (S8), 48 (S9), 36 (S10) and 50 (S11) cumulative score points (**S2 Table**). When inducing more severe VA, such differentiation using score 1 was not possible. However, score 2 and 3 efficiently reflected the duration of induced VA (MB S11 vs PES S2S3/S2S3S4 p = 0.002 and p = 0.006) (**S4 Fig**).

Quantity and duration of VA significantly increased with incremental addition of 6, 7, 8 and 10 extrastimuli as documented by arrhythmia score 3 (MB vs PES S2S3/S2S3S4 p = 0.016, p = 0.034, p = 0.01, p = 0.005). Score 2 showed more VA with MB S11 (MB vs PES S2S3/S2S3S4 p = 0.021) **(S2 Table)**. The highest rate of VA induction with the highest cumulative arrhythmia score of 50 points was observed using ten extrastimuli (MB S11, p = 0.005).

Similarly, after isoprenaline both score 2 and score 3 documented the increase of VA (p = 0.041 and p = 0.017, respectively). (**S4 Fig**).

## Discussion

The main findings of the present study are as follows: (i) the conventional PES protocol with up to 3 extrastimuli shows a low yield of VA under baseline conditions, which increases after adrenergic stimulation by isoprenaline administration, (ii) a modified PES protocol including more extrastimuli results in increased VA inducibility, (iii) the increase of VA inducibility by isoprenaline application was more efficient using the MB rather than the conventional PES protocol, and (iv) our novel arrhythmia score documents both the quantity and duration of induced VA.

Since murine *in-vivo* electrophysiology was established by Berul et al. two decades ago [17], several groups successfully performed PES in mice by using adapted protocols from humans with different stimulation protocols and definitions of VA [18, 22–30]. Particularly, the assessment of the ventricular arrhythmogenic potential in untreated wildtype mice remains challenging, due to the low yield of conventional PES protocols [30]. As previously reported and according to our data induced VA are mainly PVCs. VA can be regularly seen in various species including mice [30], rats [31] and men without disease condition [32]. The clinical significance or prognostic value of single PVC is not particularly high or at least remains unknown in human data [33, 34]. Therefore, the significance as a target VA during EPS should be judged in a measured manner in murine models. Based on these backgrounds, in the present study we have developed our new PES protocol (mini-burst [MB]) with more extrastimuli and new arrhythmia score which can also reflect duration of VA. As shown in **Fig 3**, MB protocols (particularly with MB S7 or more) enabled induction of short lasting VT (VT <1s). This result suggests that using this MB protocol more definite induction of VT, not merely PVC, may be expected in murine models with arrhythmogenic properties, resulting in the improvement of sensitivity.

Due to the difference of the action potentials of mice and humans the adequate PES protocol can be different. In the present study, we intended to investigate the availability of other PES protocols, which may enable reproducible induction of pathological VA. We have demonstrated the feasibility and accuracy of arrhythmia evaluation in mice when applying PES protocols with additional extrastimuli. We observed that inducible arrhythmias in murine models are predominantly PVCs and VTs <1s, of which a duration > 1 s is rare. In the present study the MB protocol was developed as an acute model for standardized in-depth assessment, quantification and characterization of VA in C57B16/J wildtype mice. The main target of this study was not concluding arrhythmogenic risk for transgenic or cardiovascular disease mouse models. Therefore, the determination of arrhythmogenicity with clinical significance using this MB protocol should be further investigated in the future projects. In order to study the underlying mechanisms of VA, murine electrophysiological studies offer important insights but still demand scrutiny [16, 17].

### Systematic review of literature

Most previous studies preferred coupling of two and three extrastimuli, supposedly based on experience in human clinical practice. Noteworthy, VA induction was overall rather low and ranged widely. This might be at least partly explained by the various definition of VA between studies, while most of them could not apply the definitions recommended by recent guidelines [3]. Coupling of more than three extrastimuli seemed to increase VA induction. Meanwhile, it was not clear whether adrenergic activation with isoprenaline yielded more or rather more severe VA.

### Electrophysiological study and ventricular PES

We controlled our study for a number of variables known to influence cardiac arrhythmogenicity. Thus, normal cardiac function was assured in a subgroup of tested wildtype C57BL/6J mice by cardiac echocardiography. The core body temperature of the animals was strictly controlled as it may trigger the development of calcium-dependent afterdepolarizations by changing heart rate [35] (**S1 Fig**).

Under baseline conditions, conventional PES did not succeed in VA induction, while the MB technique was effective in half of the mice tested. Mostly, single PVCs could be induced, which needs to be considered when addressing specific endpoints.

VT in structurally normal hearts can be difficult to induce in PES. In those cases, rapid burst pacing or isoprenaline infusion can be useful as indicated by previous [5, 30] and our findings. As a result of our literature review, we adjusted previous stimulation protocols in attempt to facilitate reproducible VA induction in wildtype mice with a defined protocol. The 2 second-interval between each stimulation episode was adopted from the human electrophysiological test, because our literature review revealed that data on Ca^2+^ decay after stimulation episodes in mice were scarce. In line with our hypothesis, more extrastimuli compared to conventional PES enabled effective and reproducible VA induction. In this study, the MB protocol was used as an acute model to assess VA in C57Bl6/J wildtype mice.

### Arrhythmia assessment

Arrhythmia scoring systems may help to evaluate the vulnerability for VA in given experimental models [4]. In the present study, we aimed to classify VA by their number and duration. The three currently used scoring systems differentially address the different types of VA. The previously established scores 1 and 2 focus on short runs of VA with maximum scoring points for couplets and triplets, respectively. VT is counted equally high. Here, we intended to emphasize the meaning of short runs of ventricular complexes as couplets, triplets and to integrate VT of more or less than one second. Therefore, we have designed a more detailed arrhythmia score better reflecting the duration of VA as compared to the aforementioned scores.

Compared to the arrhythmia score 2, the novel score 3 displayed a clear increase in occurrence and duration of VA by using the modified stimulation protocol. Also, an increase of VA induction could be shown after adrenergic activation by isoprenaline. These findings are consistent with triggered activity as a possible pathomechanism due to changed Ca^2+^-homeostasis in the absence of structural heart disease [11–13].

We have shown here that effectively induced VA using a modified PES protocol can be more precisely evaluated using the novel arrhythmia scoring system developed in our study. This arrhythmia score may be adopted for electrophysiological studies in mice with arrhythmogenic substrates such as ischemic cardiomyopathy and others, and eventually used to assess ventricular vulnerability. We therefore suggest introducing both the modified stimulation protocol and the novel scoring system in studies on arrhythmogenicity in the mouse heart.

### Limitations

There are some limitations in the present study. First, we did not investigate whether and how arrhythmia induction translates into long-term outcome. This is, however, beyond the aim of the present study. Here, we assessed induction of VA in order to present it as an acute model. Second, only C57BL6/J mice were examined. This does not allow translation into other strains, genetically modified mice, and models of structural/ischemic heart disease. Third, we cannot completely exclude calcium accumulation during PES. We adopted 2 seconds intervals between each stimulation episode in the present study. In the clinical setting during PES in human, in which normal heart rate is much slower as compared to mice, the interval between stimulation episodes was set at least 2 seconds traditionally [13, 20]. Louch et al. showed previously in rat that after termination of high frequency stimulation, resting intracellular calcium quickly returns to a normal steady-state condition [36]. Therefore we assume that 2 seconds of resting time between each stimulation episode is a reasonable experimental setting. Fourth, regarding VA inducibility under isoprenaline administration, the lack of statistical significance may be ascribed to the accumulation of intracellular calcium after multiple stimulation procedures. Further researches with randomized sequential orders should be conducted to exclude this possibility, i.e. VA induction with isoprenaline administration followed by VA induction without isoprenaline administration. Additionally the sequential order of MB and PES was not randomized. Higher inducibility during MB, which was always performed before PES, suggested that this inducibility was not due to the intracellular calcium accumulation. Therefore, we believe the validity of our data. Finally, we did not further distinguish between local re-entry and focal mechanisms of VA generation/perpetuation, both of which might play a central role in presented induction protocols. Optical or multi-electrode mapping might be of interest to investigate the mechanisms of induced VA and how they relate to the different stimulation protocols.

## Conclusions

A modified in-vivo PES protocol enhances VA inducibility in the mouse heart, with and without pharmacological adrenergic stimulation. Combining such a modified PES protocol with a refined scoring system, VA inducibility may be assessed more efficiently. Implementation of this approach might translate into more efficient use of laboratory animals as well as to improved experimental read-out of murine models of ventricular arrhythmias.

## Supporting information

S1 Table Echocardiographic baseline characteristics. Echocardiographic baseline characteristics for animals undergoing additional prior to electrophysiological assessments. LV = left ventricle, IVS = intraventricular septum, LVPW = left ventricular posterior wall, MV = mitral valve, AV = aortic valve.
(DOCX)

S2 Table Assessment of programmed extrastimulation. Quantity and duration of VA increased when coupling 6, 7, 8 and 10 extrastimuli as seen with arrhythmia score 3 (p = 0.016, p = 0.034, p = 0.01, p = 0.005). Score 2 was higher only with MB S11 (p = 0.021). n: number of arrhythmic events; animals: number of animals in which VA was observed.
(DOCX)

S1 Fig Heart rate of mice is decisively determined by body core temperature. Heart rate was measured in five mice at different temperature levels. Three zones low, intermediate and high were exposed. Steep change of heart rate occurred at low 32–35°C (Y = 36,34*X—739,5; p = 0.0006) and high >38°C (Y = 39,99*X—995,2; p = 0.0001) temperature, rectally measured. Within an intermediate temperature zone 35–38°C (Y = 13,21*X + 46,37; p = 0.07) the effect on heart rate was minimal.
(TIFF)

S2 Fig Programmed electrical stimulation protocol. For electrophysiological study mice underwent a structured protocol. First, the pacing threshold and the ventricular refractory period (VRP) were defined. Second, programmed electrical stimulation protocol (PES) was performed starting with the Mini-burst (MB) which was followed by the conventional PES. Intervals of 2s were allowed between the pacing maneuvers. Third, after application of isoprenaline (iso) the MB with ten extrastimuli (MB S11) was introduced followed by the conventional PES.
(TIFF)

S3 Fig Heart rate after isoprenaline injection. Adrenergic activation due to injection of isoprenaline (1μg/g i.p.) raised heart rate within 20 seconds. Effect of isoprenaline on heart rate within the first 40 seconds after injection (at time point 0 sec). Data are shown as mean with SEM, one-way ANOVA p = 0.0328 (20 sec), p = 0.0072 (23 sec), p = 0.0075 (40 sec).
(TIFF)

S4 Fig Arrhythmia scores according to PES protocols at baseline and during pharmacological adrenergic stimulation. VA induction is displayed using the novel arrhythmia score (Score 3) in comparison with previously reported arrhythmia scores by Faggioni et al. (7) (Score 1) and Van der Werf et al. (22) (Score 2). VA quantification after MB S11 extrastimulation is discriminated best using Score 2 and 3 at baseline (p = 0.002, p = 0.006) (A) as well as during adrenergic stimulation (isoprenaline) (p = 0.041, p = 0.017) (B).
(TIFF)

S1 Text Body core temperature control. Mice were placed in a supine position on a heated operating table. Body core temperature was measured via a rectal probe (T-type Pod ML312; Rectal Probe for Mice RET-3, ADInstruments). To assess influence of temperature change on heart rate a subset of 5 mice was exposed to a gradual temperature increased from 32 to 41°C over 53 ± 14 minutes. Three zones low, intermediate and high were exposed. Steep change of heart rate occurred at low 32–35°C (r = 0.3494, Y = 36,34*X—739,5; p = 0.0006) and high >38°C (r = 0.4463, Y = 39,99*X—995,2; p = 0.0001) temperature, rectally measured. Within an intermediate temperature zone 35–38°C (r = 0.09803, Y = 13,21*X + 46,37; p = 0.07) the effect on heart rate was minimal (S1 Fig). During EPS experiments body temperature was titrated to 36°C.
(DOCX)

S2 Text Echocardiography. Transthoracic Doppler high-resolution echocardiography (18–32 MHz, Vevo 2100, Visual Sonics) was performed in 6 mice under mild sedation. HR was 465 ± 41 bpm during measurements. LV function was determined by tracing end-diastolic and end-systolic area in parasternal long axis B-mode. Ejection fraction of 55.4 ± 1.4% implied normal LV function. Left ventricular wall thickness was measured in M-mode at the midventricular level. Diastolic function was assessed using the transmitral inflow Doppler in apical 4-chamber view (E/A 1.8 ± 0.3). Aortic valve flow was obtained by Doppler tracing of the LV outflow tract (2212.5 ± 173.3 mm/s). The measurements confirmed with previously published data.
(DOCX)

S1 Dataset Summarized datasheet. The table contains data from 12 animals in our experiments. The electrocardiographic parameters, electrophysiological parameters, echocardiographic parameters, and results of programmed electrical stimulation are listed.
(XLSX)
