# Supplementary material for: A modified approach for programmed electrical stimulation in mice: Inducibility of ventricular arrhythmias
Source: PLoS One. 2018 Aug 22;13(8):e0201910. doi: 10.1371/journal.pone.0201910 (PMC6104969; doi:10.1371/journal.pone.0201910)
Supplement: S1 Table — Echocardiographic baseline characteristics for animals undergoing additional prior to electrophysiological assessments. LV = left ventricle, IVS = intraventricular septum, LVPW = left ventricular posterior wall, MV = mitral valve, AV = aortic valve. (DOCX) [file pone.0201910.s001.docx]

**S1 Table. Echocardiographic baseline characteristics**

Echocardiographic baseline characteristics for animals undergoing additional prior to electrophysiological assessments. LV = left ventricle, IVS = intraventricular septum, LVPW = left ventricular posterior wall, MV = mitral valve, AV = aortic valve.

| **Echocardiographic properties** | |
| --- | --- |
| n | 6 |
| Body weight (g) | 25.3 ± 2.1 |
| Heart Rate (bpm) | 465 ± 41 |
| Cardiac Output (ml/min) | 16.0 ± 1.8 |
| Stroke Volume (µl) | 34.5 ± 1.6 |
| Ejection fraction (%) | 55.4 ± 1.4 |
| Fractional Shortening (%) | 13.2 ± 1.0 |
| LV Mass corrected (mg) | 76.1 ± 6.1 |
| Endsystolic Volume (µl) | 27.8 ± 2.1 |
| Enddiastolic Volume (µl) | 62.3 ± 3.5 |
| IVS end diastole (mm) | 0.80 ± 0.07 |
| IVS end systole (mm) | 0.92 ± 0.13 |
| LVID end diastole (mm) | 3.92 ± 0.07 |
| LVID end systole (mm) | 2.52 ± 0.20 |
| LVPW end diastole (mm) | 0.63 ± 0.09 |
| LVPW end systole (mm) | 0.97 ± 0.06 |
| MV E/A | 1.8 ± 0.3 |
| MV Deceleration time (ms) | 15.3 ± 1.8 |
| Isovolumic Contraction Time (ms) | 9.7 ± 2.7 |
| Isovolumic Relaxation Time (ms) | 15.0 ± 1.6 |
| Aortic Ejection Time (ms) | 46.8 ± 6.0 |
| AV Peak Velocity (mm/s) | 2212.5 ± 173.3 |
| AV Mean Gradient (mmHg) | 7.0 ± 1.3 |
