# Supplementary material for: A modified approach for programmed electrical stimulation in mice: Inducibility of ventricular arrhythmias
Source: PLoS One. 2018 Aug 22;13(8):e0201910. doi: 10.1371/journal.pone.0201910 (PMC6104969; doi:10.1371/journal.pone.0201910)
Supplement: S2 Table — Quantity and duration of VA increased when coupling 6, 7, 8 and 10 extrastimuli as seen with arrhythmia score 3 (p = 0.016, p = 0.034, p = 0.01, p = 0.005). Score 2 was higher only with MB S11 (p = 0.021). n: number of arrhythmic events; animals: number of animals in which VA was observed. (DOCX) [file pone.0201910.s002.docx]

**S2 Table: Assessment of programmed extrastimulation.**

Quantity and duration of VA increased when coupling 6, 7, 8 and 10 extrastimuli as seen with arrhythmia score 3 (p=0.016, p=0.034, p=0.01, p=0.005). Score 2 was higher only with MB S11 (p=0.021). n: number of arrhythmic events; animals: number of animals in which VA was observed

|  | **PES S3** | | **PES S4** | | **MB S4** | | **MB S5** | | **MB S6** | | **MB S7** | | **MB S8** | | **MB S9** | | **MB S10** | | **MB S11** | |
| --- | --- | --- | --- | --- | --- | --- | --- | --- | --- | --- | --- | --- | --- | --- | --- | --- | --- | --- | --- | --- |
|  | n | animals | n | animals | n | animals | n | animals | n | animals | n | animals | n | animals | n | animals | n | animals | n | animals |
| Affected |  | 0 |  | 0 |  | 3 |  | 5 |  | 4 |  | 5 |  | 6 |  | 6 |  | 6 |  | 6 |
| PVC | 0 | 0 | 0 | 0 | 2 | 1 | 4 | 3 | 6 | 3 | 3 | 2 | 7 | 4 | 4 | 4 | 3 | 3 | 9 | 5 |
| Couplet | 0 | 0 | 0 | 0 | 1 | 1 | 1 | 1 | 1 | 1 | 3 | 3 | 2 | 2 | 3 | 2 | 1 | 1 | 4 | 3 |
| Triplet | 0 | 0 | 0 | 0 | 1 | 1 | 1 | 1 | 3 | 2 | 1 | 1 | 1 | 1 | 1 | 1 | 1 | 1 | 1 | 1 |
| VT<1s | 0 | 0 | 0 | 0 | 2 | 2 | 4 | 2 | 1 | 1 | 6 | 4 | 4 | 3 | 5 | 2 | 4 | 3 | 5 | 2 |
| VT>1s | 0 | 0 | 0 | 0 | 0 | 0 | 0 | 0 | 0 | 0 | 0 | 0 | 1 | 1 | 1 | 1 | 1 | 1 | 0 | 0 |
| Score 1 | 0 | | 0 | | 11 | | 17 | | 14 | | 27 | | 22 | | 27 | | 20 | | 26 | |
| Score 2 | 0 | | 0 | | 17 | | 27 | | 25 | | 40 | | 37 | | 41 | | 30 | | 45* | |
| Score 3 | 0 | | 0 | | 19 | | 31 | | 26 | | 46* | | 43* | | 48* | | 36 | | 50** | |
