# Supplementary figures and images for: A modified approach for programmed electrical stimulation in mice: Inducibility of ventricular arrhythmias
Source: PLoS One. 2018 Aug 22;13(8):e0201910. doi: 10.1371/journal.pone.0201910 (PMC6104969; doi:10.1371/journal.pone.0201910)

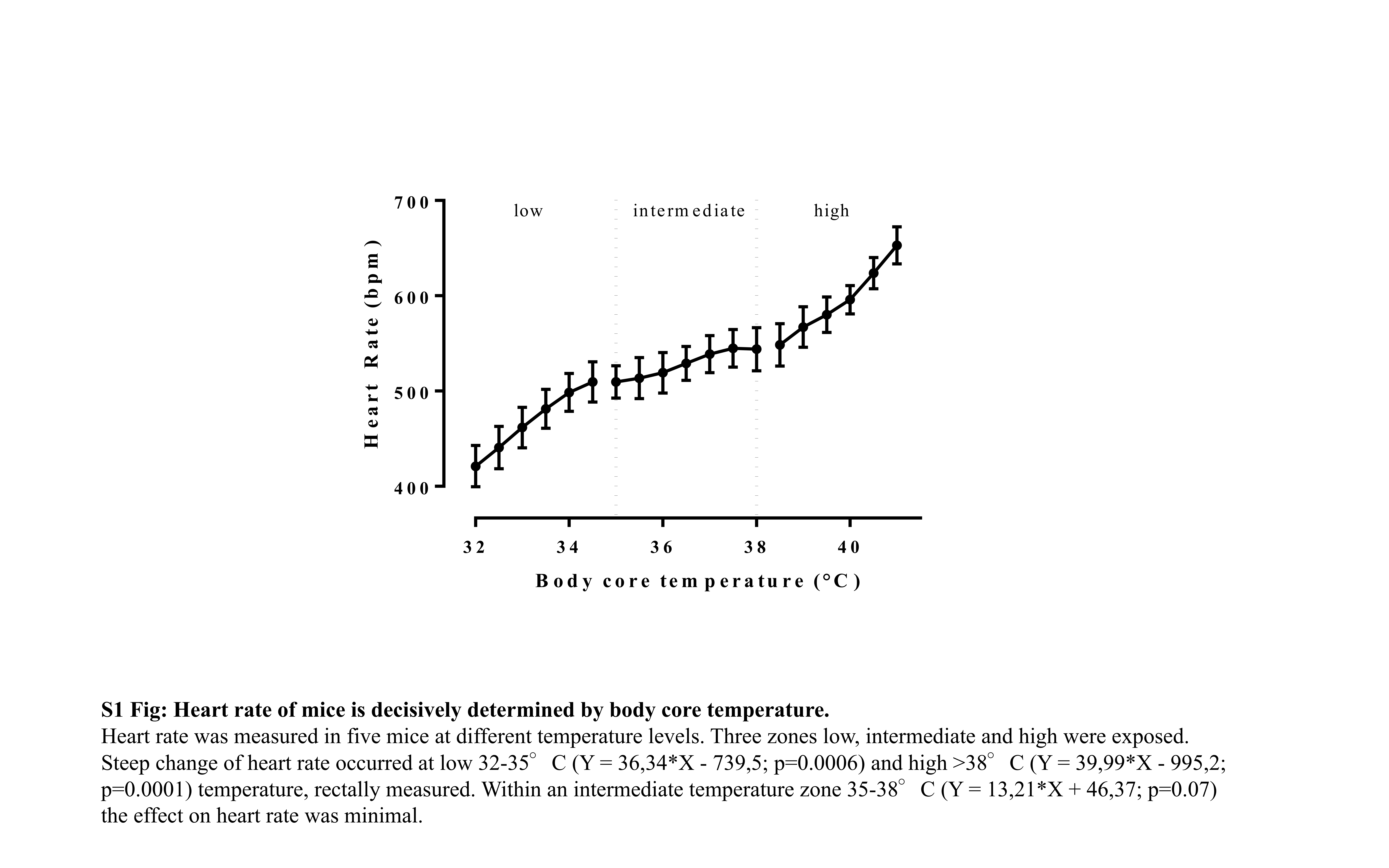

Supplement: S1 Fig — Heart rate was measured in five mice at different temperature levels. Three zones low, intermediate and high were exposed. Steep change of heart rate occurred at low 32–35°C (Y = 36,34*X—739,5; p = 0.0006) and high >38°C (Y = 39,99*X—995,2; p = 0.0001) temperature, rectally measured. Within an intermediate temperature zone 35–38°C (Y = 13,21*X + 46,37; p = 0.07) the effect on heart rate was minimal. (TIFF) [file pone.0201910.s003.tiff]

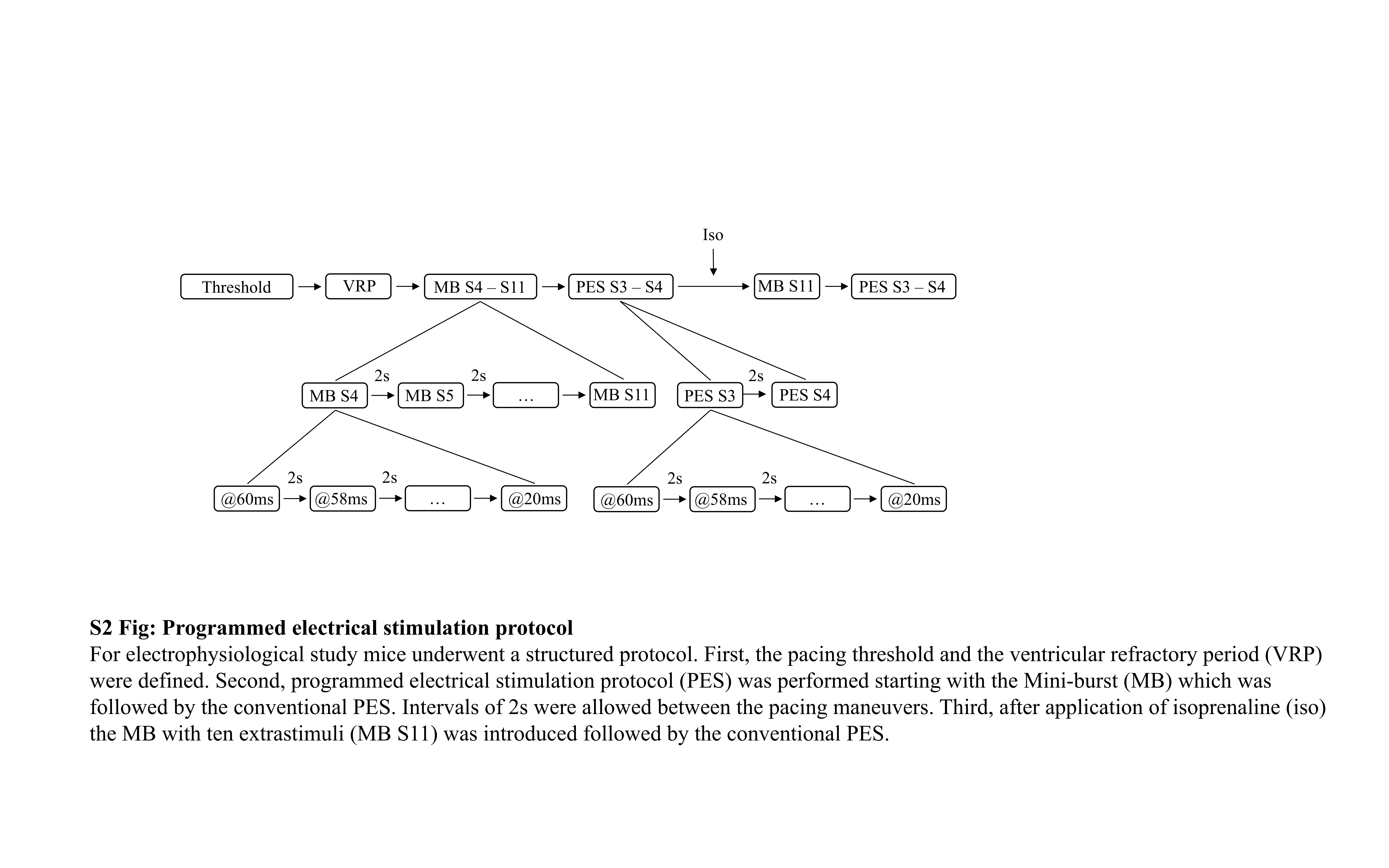

Supplement: S2 Fig — For electrophysiological study mice underwent a structured protocol. First, the pacing threshold and the ventricular refractory period (VRP) were defined. Second, programmed electrical stimulation protocol (PES) was performed starting with the Mini-burst (MB) which was followed by the conventional PES. Intervals of 2s were allowed between the pacing maneuvers. Third, after application of isoprenaline (iso) the MB with ten extrastimuli (MB S11) was introduced followed by the conventional PES. (TIFF) [file pone.0201910.s004.tiff]

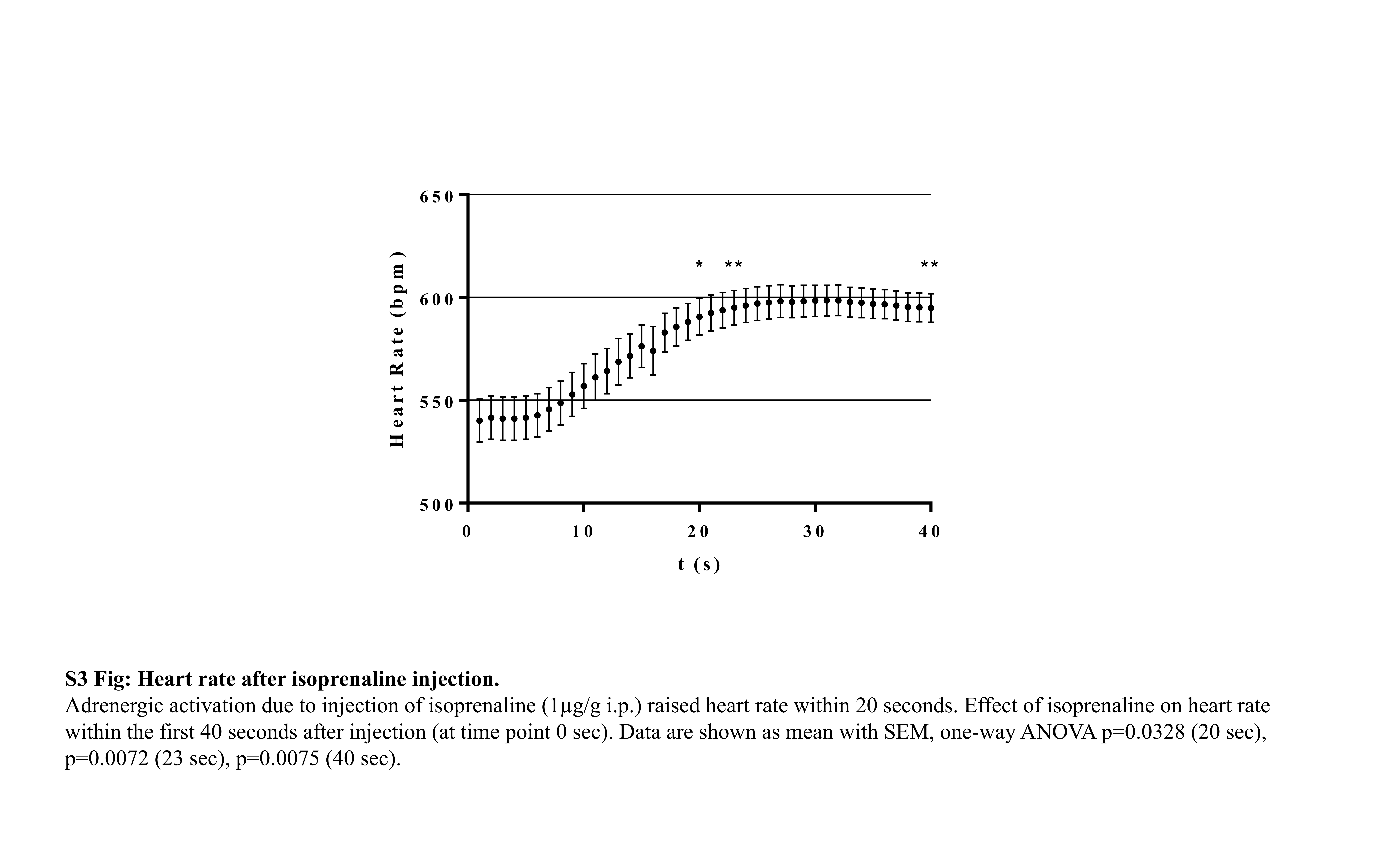

Supplement: S3 Fig — Adrenergic activation due to injection of isoprenaline (1μg/g i.p.) raised heart rate within 20 seconds. Effect of isoprenaline on heart rate within the first 40 seconds after injection (at time point 0 sec). Data are shown as mean with SEM, one-way ANOVA p = 0.0328 (20 sec), p = 0.0072 (23 sec), p = 0.0075 (40 sec). (TIFF) [file pone.0201910.s005.tiff]

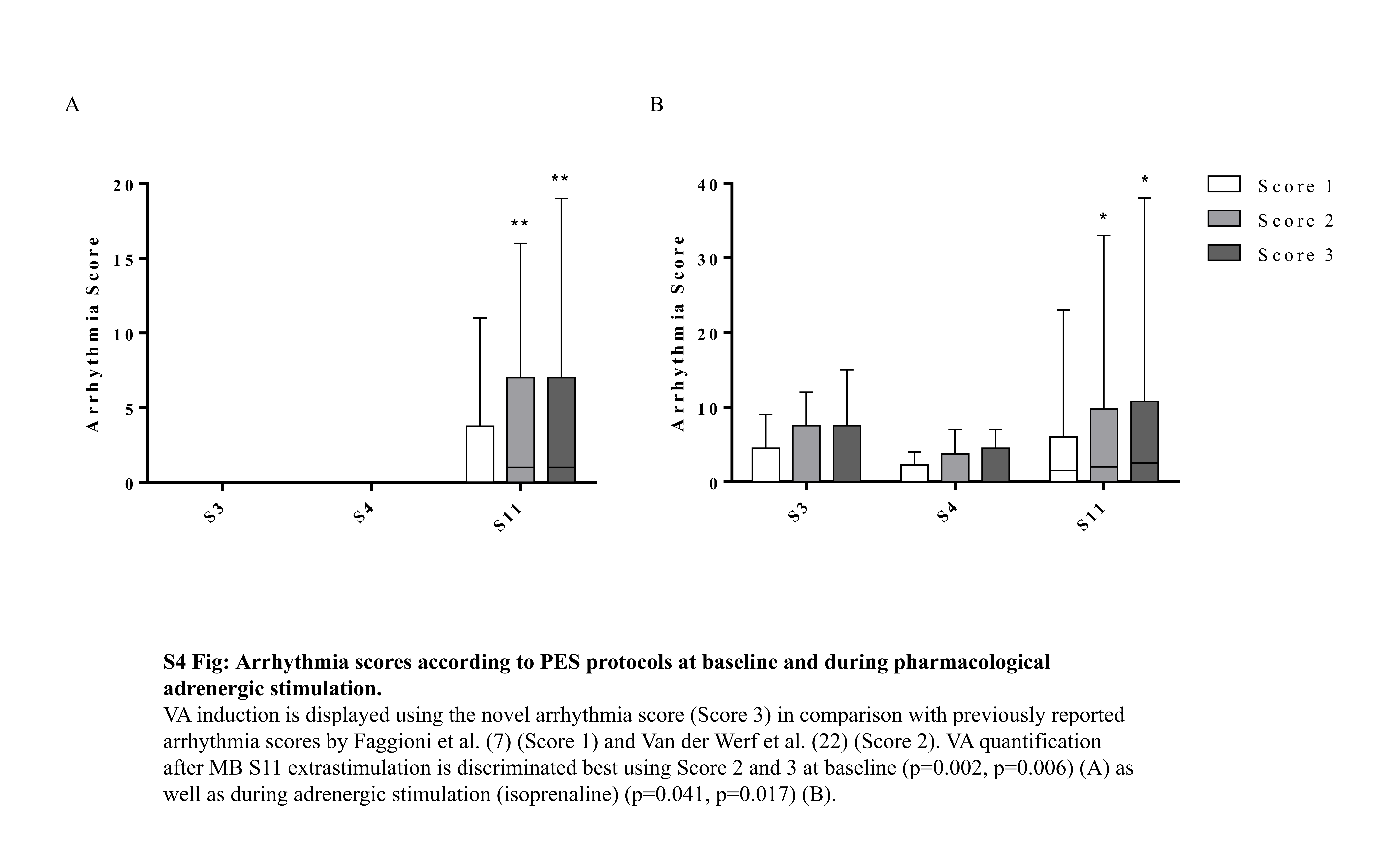

Supplement: S4 Fig — VA induction is displayed using the novel arrhythmia score (Score 3) in comparison with previously reported arrhythmia scores by Faggioni et al. (7) (Score 1) and Van der Werf et al. (22) (Score 2). VA quantification after MB S11 extrastimulation is discriminated best using Score 2 and 3 at baseline (p = 0.002, p = 0.006) (A) as well as during adrenergic stimulation (isoprenaline) (p = 0.041, p = 0.017) (B). (TIFF) [file pone.0201910.s006.tiff]
