# Supplementary material for: A modified approach for programmed electrical stimulation in mice: Inducibility of ventricular arrhythmias
Source: PLoS One. 2018 Aug 22;13(8):e0201910. doi: 10.1371/journal.pone.0201910 (PMC6104969; doi:10.1371/journal.pone.0201910)
Supplement: S1 Text — Mice were placed in a supine position on a heated operating table. Body core temperature was measured via a rectal probe (T-type Pod ML312; Rectal Probe for Mice RET-3, ADInstruments). To assess influence of temperature change on heart rate a subset of 5 mice was exposed to a gradual temperature increased from 32 to 41°C over 53 ± 14 minutes. Three zones low, intermediate and high were exposed. Steep change of heart rate occurred at low 32–35°C (r = 0.3494, Y = 36,34*X—739,5; p = 0.0006) and high >38°C (r = 0.4463, Y = 39,99*X—995,2; p = 0.0001) temperature, rectally measured. Within an intermediate temperature zone 35–38°C (r = 0.09803, Y = 13,21*X + 46,37; p = 0.07) the effect on heart rate was minimal (S1 Fig). During EPS experiments body temperature was titrated to 36°C. (DOCX) [file pone.0201910.s007.docx]

**S1 Text: Body core temperature control**

Mice were placed in a supine position on a heated operating table. Body core temperature was measured via a rectal probe (T-type Pod ML312; Rectal Probe for Mice RET-3, ADInstruments). To assess influence of temperature change on heart rate a subset of 5 mice was exposed to a gradual temperature increased from 32 to 41°C over 53 ± 14 minutes. Three zones low, intermediate and high were exposed. Steep change of heart rate occurred at low 32-35°C (r=0.3494, Y = 36,34*X - 739,5; p=0.0006) and high >38°C (r=0.4463, Y = 39,99*X - 995,2; p=0.0001) temperature, rectally measured. Within an intermediate temperature zone 35-38°C (r=0.09803, Y = 13,21*X + 46,37; p=0.07) the effect on heart rate was minimal (**S1 Fig**). During EPS experiments body temperature was titrated to 36°C.
