# Supplementary material for: A modified approach for programmed electrical stimulation in mice: Inducibility of ventricular arrhythmias
Source: PLoS One. 2018 Aug 22;13(8):e0201910. doi: 10.1371/journal.pone.0201910 (PMC6104969; doi:10.1371/journal.pone.0201910)
Supplement: S2 Text — Transthoracic Doppler high-resolution echocardiography (18–32 MHz, Vevo 2100, Visual Sonics) was performed in 6 mice under mild sedation. HR was 465 ± 41 bpm during measurements. LV function was determined by tracing end-diastolic and end-systolic area in parasternal long axis B-mode. Ejection fraction of 55.4 ± 1.4% implied normal LV function. Left ventricular wall thickness was measured in M-mode at the midventricular level. Diastolic function was assessed using the transmitral inflow Doppler in apical 4-chamber view (E/A 1.8 ± 0.3). Aortic valve flow was obtained by Doppler tracing of the LV outflow tract (2212.5 ± 173.3 mm/s). The measurements confirmed with previously published data. (DOCX) [file pone.0201910.s008.docx]

**S2 Text: Echocardiography**

Transthoracic Doppler high-resolution echocardiography (18-32 MHz, Vevo 2100, Visual Sonics) was performed in 6 mice under mild sedation^1,2^. HR was 465 ± 41 bpm during measurements. LV function was determined by tracing end-diastolic and end-systolic area in parasternal long axis B-mode. Ejection fraction of 55.4 ± 1.4 % implied normal LV function. Left ventricular wall thickness was measured in M-mode at the midventricular level. Diastolic function was assessed using the transmitral inflow Doppler in apical 4-chamber view (E/A 1.8 ± 0.3). Aortic valve flow was obtained by Doppler tracing of the LV outflow tract (2212.5 ± 173.3 mm/s). The measurements confirmed with previously published data^2^.

**References**

1. Merx MW, Gorressen S, van de Sandt AM, Cortese-Krott MM, Ohlig J, Stern M, et al. Depletion of circulating blood NOS3 increases severity of myocardial infarction and left ventricular dysfunction. Basic Res Cardiol. 2014;109:398 doi:10.1007/s00395-013-0398-1

2. Erkens R, Kramer CM, Luckstadt W, Panknin C, Krause L, Weidenbach M, et al. Left ventricular diastolic dysfunction in Nrf2 knock out mice is associated with cardiac hypertrophy, decreased expression of SERCA2a, and preserved endothelial function. Free Radic Biol Med. 2015;89:906-917 doi:10.1016/j.freeradbiomed.2015.10.409
